# Digital adherence technologies to improve tuberculosis treatment outcomes in China: a cluster-randomised superiority trial

**DOI:** 10.1016/S2214-109X(23)00068-2

**Published:** 2023-04-14

**Authors:** Xiaoqiu Liu, Jennifer Thompson, Haiyan Dong, Sedona Sweeney, Xue Li, Yanli Yuan, Xiaomeng Wang, Wangrui He, Bruce Thomas, Caihong Xu, Dongmei Hu, Anna Vassall, Shitong Huan, Hui Zhang, Shiwen Jiang, Katherine Fielding, Yanlin Zhao

**Affiliations:** a National Center for TB Control and Prevention, Chinese Center for Disease Control and Prevention, Beijing, China; b Department of Infectious Disease Epidemiology, London School of Hygiene & Tropical Medicine, London, UK; c Department of Global Health and Development, London School of Hygiene & Tropical Medicine, London, UK; d PATH China Office, Shanghai, China; e Jilin Provincial Center for Disease Control and Prevention, Changchun, China; f Zhejiang Provincial Center for Disease Control and Prevention, Hangzhou, China; g Jiangxi Provincial Center for Disease Control and Prevention, Nanchang, China; h Arcady Group, Richmond, VA, USA; i Bill & Melinda Gates Foundation China Office, Beijing, China

## Abstract

**Background:**

Drug-sensitive tuberculosis treatment requires 6 months of therapy, so adherence problems are common. Digital adherence technologies might improve tuberculosis treatment outcomes. We aimed to evaluate the effect of a daily reminder medication monitor, monthly review of adherence data by the health-care provider, and differentiated care for patients with adherence issues, on tuberculosis treatment adherence and outcomes.

**Methods:**

We did a cluster-randomised superiority trial across four prefectures in China. 24 counties or districts (clusters) were randomly assigned (1:1) to intervention or control groups. We enrolled patients aged 18 years or older with GeneXpert-positive, rifampicin-sensitive pulmonary tuberculosis, who were receiving daily fixed-dose combination treatment. Patients in the intervention group received a medication monitor for daily drug-dosing reminders, monthly review of adherence data by health-care provider, and management of poor adherence; and patients in the control group received routine care (silent-mode monitor-measured adherence). Only the independent endpoints review committee who assessed endpoint data for some participants were masked to study group assignment. Patients were followed up (with sputum solid culture) at 12 and 18 months. The primary outcome was a composite of death, loss to follow-up, treatment failure, switch to multidrug-resistant tuberculosis treatment, or tuberculosis recurrence by 18 months from treatment start, analysed in the intention-to-treat population. Analysis accounted for study design with multiple imputation for the primary outcome. This trial is now complete and is registered with ISRCTN, 35812455.

**Findings:**

Between Jan 26, 2017, and April 3, 2019, 15 257 patients were assessed for eligibility and 3074 were enrolled, 2686 (87%) of whom were included in the intention-to-treat population. 1909 (71%) of 2686 patients were male, 777 (29%) were female, and the median age was 44 years (IQR 29–58). By 18 months from treatment start, using multiple imputation for missing outcomes, 239 (16% [geometric mean of cluster-level proportion]) of 1388 patients in the control group and 224 (16%) of 1298 in the intervention group had a primary composite outcome event (289 [62%] of 463 events were loss to follow-up during treatment and 42 [9%] were tuberculosis recurrence). The intervention had no effect on risk of the primary composite outcome (adjusted risk ratio 1·01, 95% CI 0·73–1·40).

**Interpretation:**

Our digital medication monitor intervention had no effect on unfavourable outcomes, which included loss to follow-up during treatment, tuberculosis recurrence, death, and treatment failure. There was a failure to change patient management following identification of treatment non-adherence at monthly reviews. A better understanding of adherence patterns and how they relate to poor outcomes, coupled with a more timely review of adherence data and improved implementation of differentiated care, may be required.

**Funding:**

Bill & Melinda Gates Foundation.

## Introduction

There were an estimated 10·6 million new cases of tuberculosis globally in 2021.^1^ Declines in tuberculosis incidence and deaths have been observed over the past two decades; however, these declines are unlikely to be fast enough to reach reduction milestones for 2030, and data suggest that there was an increase in 2020–21 due to the COVID-19 pandemic. China is among eight countries that contribute two-thirds of the global tuberculosis total, although, compared with 2015, by 2021 there had been a 15% reduction in tuberculosis incidence (from 65 cases per 100 000 population) and a 25% reduction in mortality (from two deaths per 100 000 population).^1^

National guidelines recommend daily fixed-dose combination therapy to treat drug-sensitive tuberculosis, consisting of 2 months of isoniazid, rifampicin, pyrazinamide, and ethambutol, followed by 4 months of isoniazid and rifampicin. Treatment adherence is considered important for cure and reducing recurrence of tuberculosis.^2^, ^3^

A major component of the directly observed treatment short-course strategy, introduced in China in 1992 and with national coverage by 2005, is directly observed therapy, defined as direct observation of treatment usually by a health-care worker or treatment supporter, to help improve medication adherence. However, a systematic review of treatment support using studies from China showed that only 20% of patients had directly observed therapy by a health professional and more than 50% of patients were self-administering treatment.^4^ Despite this, China's treatment success for new and relapsed drug-sensitive tuberculosis is reported to be 95%.^1^

**Research in context**

**Evidence before this study**

We searched Medline and Embase in December, 2015, for papers published from Jan 1, 2000, to Dec 1, 2015, with no language restrictions, using the terms (“digital pill box*” OR “smart pill box*” OR “SMS” OR “text messag*”) AND “TB” OR “tuberculosis”. We found one systematic review assessing the effect of mobile phone text messaging on treatment adherence used as a proxy for treatment outcomes and development of drug resistance. Four studies (three observational cohort studies and one randomised trial) were included in the review, meta-analysis was not conducted, and the authors concluded that there were mixed findings for the effectiveness of text messaging to promote adherence to tuberculosis treatment. Our previous study in China, published in 2015, reported improved adherence to tuberculosis treatment in patients who received text messaging or smart pill box reminders compared with those who did not. The study was not powered to evaluate treatment outcomes. Since 2019, three studies have reported improved tuberculosis outcomes using digital treatment adherence technologies. A study in Kenya assessed weekly motivational messages, daily text message reminders, an unstructured supplementary service data platform for patients to confirm daily adherence followed by text message and calls from the research team for patients who had not confirmed adherence, and clinic notification of patients with no confirmation for more than 2 days. The intervention reduced the risk of poor treatment outcomes (on-treatment death, loss to follow-up, or treatment failure) by 68%, entirely through reducing loss to follow-up. A stepped-wedge trial in Uganda assessed a text-message-based intervention, where patients received daily text message dosing reminders and were asked to confirm a dose taken using a toll-free phone number. Adherence data were reviewed at clinics visits every 2 weeks or monthly and resulted in differentiated management. The authors showed improved successful treatment outcomes (defined as cured or completed treatment) with the intervention, although only among a per-protocol population (which included 97% of patients in the control phase and 52% in the intervention phase) who enrolled within the first 2 months of treatment. In Peru, a per-protocol analysis of an individually randomised trial showed higher treatment success (cured or completed treatment) among patients who had a real-time medication event reminder monitor versus those who received standard of care. A systematic review in 2022 reported variable effects of digital adherence technologies on tuberculosis treatment outcomes.

**Added value of this study**

To our knowledge, this is the first trial to evaluate the effect of a digital adherence technology intervention (smart pill box reminder, monthly review of adherence data, and differentiated care for patients with adherence issues) on a composite outcome of death, loss to follow-up, treatment failure, or subsequent retreatment including culture-confirmed recurrence, among patients with drug-sensitive tuberculosis. We found that the intervention was not adequate to influence poor treatment outcomes, in particular loss to follow-up or tuberculosis recurrence. There was a failure to change patient management following identification of non-adherence at the monthly reviews. We did, however, show a reduction in treatment non-adherence in the intervention group compared with the standard of care group, similar to in our previous study, indicating that the smart pill box intervention improved treatment adherence.

**Implications of all the available evidence**

There is no strong evidence that digital adherence technology interventions improve treatment outcomes, including incidence of tuberculosis recurrence, among patients with drug-sensitive tuberculosis. More frequent review of adherence data, with a streamlined approach for identifying patients with adherence issues and escalating supportive management of these patients, might be key to improving tuberculosis treatment outcomes.

Digital adherence technologies, including SMS and electronic pill boxes, which support patients in their adherence to treatment, have the potential to enhance patient care through improving interactions between patients and health-care providers, and improving treatment adherence and outcomes.^5^, ^6^ Electronic pill boxes usually involve a daily audio or visual reminder and box opening (considered a proxy for dose taken), which is digitally recorded and then used by the health-care provider either in real time or at routine visits to initiate more adherence support for patients who are having problems with adherence (referred to as differentiated care). SMS interventions include patients receiving a daily SMS reminder, sometimes combined with patients sending a text message to the health-care provider to confirm a dose has been taken. WHO's drug-sensitive tuberculosis updated treatment guidelines made a conditional recommendation, with very low certainty of evidence, for tracers (such as mobile phone SMS) or digital medication monitors to be offered to patients with tuberculosis.^7^

Three studies have shown improved tuberculosis treatment outcomes with digital adherence technologies. An individually randomised trial in Kenya showed a reduction in poor outcomes (on-treatment death, loss to follow-up, or treatment failure), mainly through reducing loss to follow-up, when using SMS reminders and an unstructured supplementary service data intervention.^8^ Treatment success (defined as cured or completed treatment) was increased in a stepped-wedge trial in Uganda, in a per-protocol population of patients who received the SMS-style (99DOTS) intervention versus community-based directly observed therapy.^9^ The 99DOTS intervention included daily automated SMS dosing reminders and patients confirming doses taken daily. In Peru, a per-protocol analysis of an individually randomised trial showed an increase in treatment success (cured or completed treatment) among patients who received a real-time medication event reminder monitor, where patients received SMS reminders if the monitor was not opened at the scheduled treatment time, escalating to sending an SMS to a previously designated relative or treatment supporter if the monitor remained unopened.^10^

We aimed to evaluate the effect of a daily reminder medication monitor, monthly review of adherence data by the health-care provider, and differentiated care for patients with adherence issues, on tuberculosis treatment adherence and outcomes.

## Methods

### Study design

We did a cluster-randomised superiority trial in four prefectures (administrative subdivisions of provinces) in China. Geographical areas served by a tuberculosis dispensary or designated hospital were the unit of randomisation (clusters).^11^ Patients were assigned to clusters according to the dispensary or designated hospital where they received their tuberculosis treatment. The trial was approved by the Institutional Review Board of the Chinese Center for Disease Control and Prevention (CDC) and the London School of Hygiene & Tropical Medicine Ethics Committee.

### Participants

Prefectures were initially screened to identify at least five counties or districts with more than 300 patients with pulmonary tuberculosis in 2014 (to achieve our sample size), access to GeneXpert testing and culture diagnosis, tuberculosis services supplied by a designated hospital or dispensary, and use of a daily tuberculosis treatment regimen. Consent was obtained at the provincial and prefecture-level CDC and from the local health authority of each cluster. We enrolled consecutive patients aged 18 years or older with GeneXpert-positive and rifampicin-sensitive pulmonary tuberculosis, who were receiving daily fixed-dose combination treatment and were able to attend follow-up visits at 12 and 18 months after treatment start. Patients provided written informed consent to participate.

### Randomisation and masking

Constrained randomisation was used to randomly assign 24 clusters (1:1) to intervention or control groups, balanced for prefecture (difference by group was at most one prefecture), health setting type (hospital or dispensary; seven hospitals and five dispensaries in each group), area (urban or rural; difference in number per group was at most one), and sputum smear-positive tuberculosis notifications in 2015 (difference in average notifications by group was at most ten cases). Randomisation was done by the trial statistician using Stata (version 14). After randomisation, it was identified that two clusters in the intervention group used the same dispensary, so these were combined into one cluster.

Cluster randomisation was justified to reduce contamination between groups and for logistical convenience: the intervention required changes to the delivery of care, so there were concerns that individual randomisation would lead to staff changing their behaviour towards patients in the control group and patients discussing care with one another.

Only the independent endpoints review committee who assessed endpoint data for some participants were masked to study group assignment.

### Procedures

In all clusters, the tuberculosis doctors and relevant staff from the county-level CDC received a 3-day training on enrolment of patients, data collection, and the medication event reminder monitor. In both groups, patients were given a daily tuberculosis treatment regimen of 2 months of isoniazid, rifampicin, pyrazinamide, and ethambutol, followed by 4 months of isoniazid and rifampicin, and a medication event reminder monitor to store medication. The medication event reminder monitor recorded the dates and times that it was opened for more than 2 s for patients to take their medication, and recorded a daily output to indicate that it was working. Further training was conducted after the start of the study on quality control of data and follow-up of participants after the end of treatment.

In clusters in the intervention group, the tuberculosis doctors at the county level received a 1·5-day training on delivering the intervention and doctors at the township and village level received a half-day training on the intervention. The medication event reminder monitor was set up to give an audio and visual reminder to take medication (daily, three times within 5 min) and attend monthly clinic visits. The default time for the daily dosing reminder was 0800 h but could be changed to an alternative time in the morning according to patient preferences. At clinic visits, the doctor could display monitor openings in the previous month on their computer, to discuss adherence with the patient. Intensive management was initiated at the first instance of non-adherence of 20–50% of treatment days when monitor was not opened as a proxy for doses missed in the previous month; township doctors would be asked to visit patients every 2 weeks and village doctors every week. At the second instance of non-adherence of 20–50% or the first instance of non-adherence of greater than 50% in the previous month, patients were switched to directly observed therapy, with medical staff observing therapy administration. Every month, staff from China CDC or the county-level CDC visited each intervention cluster to check the intervention was being implemented as planned.

Patients in control clusters had the reminder functions on the medication event reminder monitor disabled. Managing doctors could not review monitor openings at clinic visits, but data were collected for the trial. In consultation with the doctor, patients chose whether to take medication under direct observation by a health-care worker, family member, or through self-administration.

Patients attended monthly routine clinic visits for the 6 months of treatment. Patients had routine sputum collection for smear microscopy at 2 months, 5 months, and end of treatment. At end of treatment, an additional sputum sample was collected for solid culture (using routine laboratories) and a chest radiograph was done to help define treatment failure. Patients were telephoned at 9 and 15 months after the start of treatment and self-reported retreatment for tuberculosis. At 12 and 18 months after the start of treatment, patients attended the clinic to provide sputum for culture, have a chest radiograph, and self-report tuberculosis retreatment.

Missed doses were measured by days with no opening recorded on the medication event reminder monitor, excluding days when patients reported not using the monitor because of travel or hospitalisation or when there was no medication event reminder monitor daily output to show it was working.

### Outcomes

The primary outcome was a composite of death, loss to follow-up (or stopping treatment due to an adverse reaction or refusal of treatment), treatment failure, switch to multidrug-resistant tuberculosis treatment, or tuberculosis recurrence by 18 months from treatment start. Treatment outcomes were based on standard national tuberculosis programme outcomes documented on the tuberculosis register (appendix pp 2–3). Recurrence was defined as a single positive culture, chest radiograph satisfying the case definition for new active tuberculosis, or self-report of retreatment. Recurrences identified by chest radiograph or self-report were reviewed by an independent endpoint review committee masked to study group assignment. This composite outcome aligns with outcomes used in previous tuberculosis treatment trials.^12^, ^13^

Secondary outcomes were poor treatment outcome (death, treatment failure, loss to follow-up during treatment, and switch to multidrug-resistant tuberculosis treatment), loss to follow-up during treatment, poor treatment outcome or recurrence within 12 months of treatment start, time to recurrence in those who had been cured or completed treatment, and 2-month smear conversion to negative among those with a positive smear at the start of treatment. Secondary outcomes for adherence were percentage of months in which patients missed at least 20% of doses, percentage of overall doses missed, and visits attended on schedule. Process measures included inability to use the fixed dose combination treatment, number of visits by township and village doctor, medication event reminder monitor malfunctions, and withdrawal from using the medication event reminder monitor. In the intervention group, we also summarised the number of times the alarm sounded each day, and change of management due to non-adherence.

Visits were defined as being attended on schedule if the number of days between visits was the same or fewer than the number of days’ worth of medication given to patients at their previous visit.

### Statistical analysis

In our original sample size calculations, 12 clusters per group and a harmonic mean of 125 patients per cluster gave 85% power to detect a 40% risk reduction in the primary composite outcome at the 5% level, assuming an 18% risk of the primary composite outcome in the control group, 5% loss to follow-up, and a coefficient of variation of outcome of 0·3. In recalculation after two clusters were combined, a harmonic mean of 108 patients per cluster (due to slower than expected recruitment), allowing for 10% loss to follow-up, gave 83% power to detect a 40% risk reduction in the primary composite outcome. Sample size calculations were conducted using the Stata command clustersampsi.

The intention-to-treat population was defined as participants enrolled into the trial, excluding those who met a post-enrolment exclusion criterion (participants who stopped taking the fixed-dose combination treatment within the first month due to an adverse reaction; permanently stopped their treatment within the first month because of travel or hospitalisation; had their treatment extended due to updated diagnosis of tuberculosis pleurisy, or tracheal or bronchial tuberculosis; or had diagnosis of drug resistance due to non-rifampicin drug resistance), and participants with a change of diagnosis confirmed by the endpoint review committee. The per-protocol population further excluded participants who withdrew early from use of the medication event reminder monitor, regardless of the reason given.

All analyses were done in Stata (version 15), using the clan command. Our primary estimand was a risk ratio (RR), calculated using the logarithm of the cluster proportions: this estimates the ratio of geometric means of the cluster-level risks in each group, so all percentages reported are geometric means. The primary analysis was adjusted for age, sex, occupation, migrant status, distance to clinic, education level, household expenditure, and smear result at treatment initiation, using the two stage approach.^14^

For the primary outcome, the primary analysis was based on multiple imputation in the intention-to-treat population. Multiple imputation due to missing composite outcome was applied with 25 imputations (appendix pp 3–4).^15^ Complete case analyses for the intention-to-treat and per-protocol populations were also done. All secondary outcomes and subgroups were analysed using complete cases only. For the primary outcome using the intention-to-treat population, prespecified subgroup complete case analyses were done for area of residence (urban or rural), clinic type, age, education level, sex, and household expenditure, and post-hoc subgroup analyses were done for smear status and GeneXpert cycle threshold. All analyses compared outcomes, measured at the individual-level, in the intervention group versus the control group.

This trial is registered with ISRCTN, 35812455.

### Role of the funding source

SH is employed by the funder of the study and contributed to the design and conduct of the trial and the writing of this manuscript. The funder of the study had no role in the decision to submit the results for publication.

## Results

**Figure 1:**
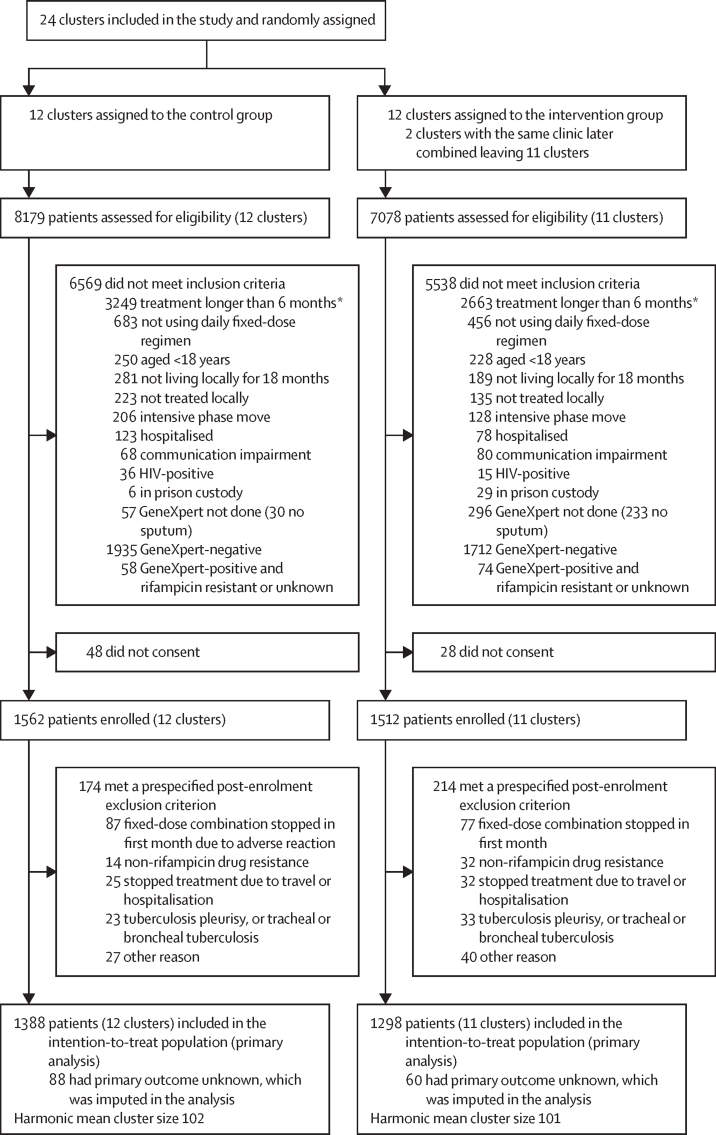
Trial profile Participants could have met more than one exclusion criterion. *Extrapulmonary tuberculosis, diabetes, or silicosis.

Between Jan 26, 2017, and April 3, 2019, 15 257 patients were assessed for eligibility and 3074 were enrolled (figure 1). In the control group, 8179 patients were screened and 6569 were excluded due to ineligibility, largely due to patients requiring treatment for more than 6 months (n=3249) or a negative or rifampicin-resistant GeneXpert result (n=1993), and 48 did not provide consent. In the intervention group, 7078 patients were screened and 5538 were excluded due to ineligibility, again mostly due to requiring treatment for more than 6 months (n=2663) or a negative or rifampicin-resistant GeneXpert result (n=1786), and 28 did not provide consent. 174 patients in the control group and 214 in the intervention group met a post-enrolment exclusion criterion and were excluded from the intention-to-treat population. 2686 patients (1388 in the control group and 1298 in the intervention group) were included in the intention-to-treat population. Follow-up continued until Oct 21, 2020. Of 23 clusters enrolled (seven in the Ganzhou prefecture, six in Hangzhou, three in Jilin, and seven in Wenzhou), 14 treated patients in tuberculosis hospitals and 17 were in rural areas (table 1).

**Table 1:** Baseline characteristics

		**Control group**	**Intervention group**
**Cluster-level covariates**			
Total clusters		12	11
Prefecture			
	Ganzhou	4 (33%)	3 (27%)
	Hangzhou	3 (25%)	3 (27%)
	Jilin	2 (17%)	1 (9%)
	Wenzhou	3 (25%)	4 (36%)
Centre type			
	Hospital	7 (58%)	7 (64%)
	Tuberculosis dispensary	5 (42%)	4 (36%)
Area type			
	Rural area	9 (75%)	8 (73%)
	Urban area	3 (25%)	3 (27%)
Number of sputum smear-positive tuberculosis cases notified in 2015		148 (94–235)	145 (113–182)
**Participant-level covariates**			
Total participants		1388	1298
Sex			
	Male	989 (71%)	920 (71%)
	Female	399 (29%)	378 (29%)
Age, years		45 (29–59)	42 (29–57)
Marital status			
	Single	299 (22%)	272 (21%)
	First marriage	990 (71%)	965 (74%)
	Other	99 (7%)	61 (5%)
Employment status			
	Unemployed (including students and retired)	261/1387 (19%)	194/1282 (15%)
	Farmer	798/1387 (58%)	585/1282 (45%)
	Other	328/1387 (24%)	503/1282 (39%)
Highest education level completed			
	None	126 (9%)	99 (8%)
	Primary school	404 (29%)	359 (28%)
	Junior middle school	494 (36%)	500 (39 %)
	High school	205 (15%)	218 (17%)
	University	159 (11%)	122 (9%)
Residency status			
	Local resident	1088 (78%)	883 (68%)
	Temporary resident	300 (22%)	415 (32%)
Household expenditure, CNY			
	<1000	182 (13%)	77 (6%)
	1001–3000	644 (46%)	741 (57%)
	≥3001	562 (40%)	480 (37%)
Smear status			
	Smear-positive	858 (62%)	817 (63%)
	Smear-negative	530 (38%)	481 (37%)
Supervision of treatment			
	Directly observed therapy*	138 (10%)	NA
	Family member supervision	307 (22%)	NA
	Self-administered	943 (68%)	NA

Data are n, n (%), n/N (%), or median (IQR). Percentages are overall, ignoring clustering. CNY=Chinese yuan renminbi. NA=not applicable.

* By health-care worker.

1909 (71%) of 2686 patients were male, 777 (29%) were female, the median age was 44 years (IQR 29–58), and 1675 (62%) had sputum smear-positive tuberculosis (table 1; appendix pp 5–6). Employment as a farmer was more common in the control group (798 [58%] of 1387 patients) than in the intervention group (585 [45%] of 1282), and patients in the control group were more likely to be local residents (defined as formally registered in the prefecture) than those in the intervention group (1088 [78%] *vs* 883 [68%]). Patients who were not local residents were living in the prefecture temporarily (eg, for work or attending university). 88 (6%) of 1388 patients in the control group and 60 (5%) of 1298 in the intervention group had missing primary composite outcome data at 18 months (appendix p 7).

Using multiple imputation for missing outcomes, 239 (16%; geometric mean of cluster-level event rate based on mean of 25 imputations) of 1388 patients in the control group and 224 (16%) of 1298 in the intervention group had a primary composite outcome event. There was no evidence of a difference in the risk of the primary composite outcome between groups (adjusted RR 1·01, 95% CI 0·73 to 1·40, p=0·95; adjusted risk difference 0·7%, 95% CI –4·5 to 5·9%). Results were similar in unadjusted, complete case, and per-protocol analyses (table 2; appendix pp 7–8).

**Table 2:** Primary and secondary outcomes, excluding adherence

	Control group (n=1388)*	Intervention group (n=1298)*	**Unadjusted RR (95% CI)**	**Adjusted RR (95% CI)**
**Primary outcome**				
Death, loss to follow-up, treatment failure, switch to multidrug-resistant tuberculosis treatment, or recurrence in 18 months†	239/1388 (16%)	224/1298 (16%)	0·99 (0·66–1·48); p=0·96	1·01 (0·73–1·40); p=0·95
**Secondary outcomes**				
Death, loss to follow-up, treatment failure, switch to multidrug-resistant tuberculosis treatment	203/1350 (14%)	188/1283 (13%)	0·94 (0·61–1·45)	0·97 (0·69–1·35)
Loss to follow-up during treatment	156/1350 (10%)	133/1283 (9%)	0·90 (0·54–1·50)	0·92 (0·59–1·44)
Death, loss to follow-up, treatment failure, switch to multidrug-resistant tuberculosis treatment, or recurrence in 12 months	215/1322 (15%)	204/1240 (15%)	1·00 (0·68–1·49)	1·03 (0·75–1·41)
Time to recurrence, rate per 100 person-years‡	14/1147 (1·5)	28/1095 (2·4)	1·60 (0·75–3·42)	..
Conversion to negative smear after 2 months§	689/759 (89%)	639/729 (89%)	1·00 (0·91–1·08)	0·99 (0·92–1·06)

Data are n/N (%) or RR (95% CI); p value unless otherwise stated. All analyses were in the intention-to-treat population. All comparisons are intervention group versus control group. RR=risk ratio.

* Number of events in number of participants, ignoring cluster; percentages are the geometric mean of the cluster-level risk or rate of an event.

† Calculated using multiple imputation; values shown by group are the imputation-mean of total number of events and geometric mean of cluster-level proportion of events; all other outcomes used complete cases.

‡ Rate difference reported between the groups in those with a successful treatment outcome; adjusted analysis not completed because of the small number of events.

§ In those with a positive smear at the start of treatment.

**Figure 2:**
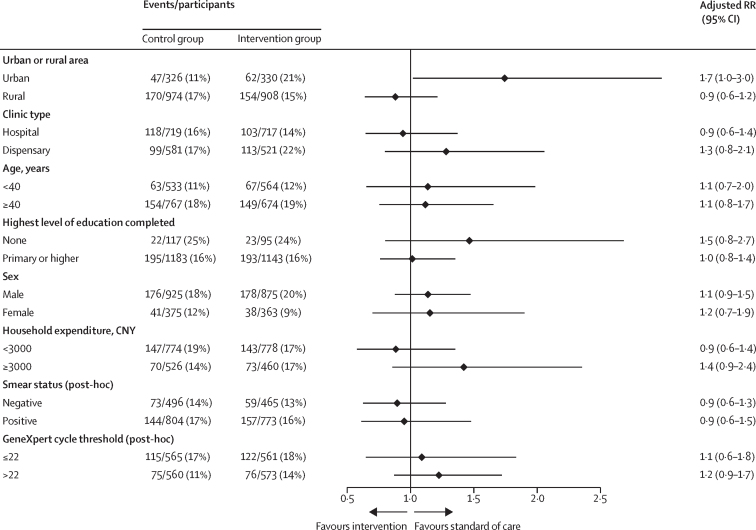
Subgroup analyses of primary composite outcome in the intention-to-treat population CNY=Chinese yuan renminbi. RR=risk ratio.

Prespecified subgroup analysis did not show significant differences in the risk of the primary composite outcome between groups by clinic type, patient age, education level, sex, or household expenditure (figure 2). The intervention was associated with increased risk of the primary composite outcome in clusters in urban areas (47 [11%; geometric mean] of 326 patients had composite outcome events in the control group *vs* 62 [21%] of 330 in the intervention group; adjusted RR 1·74, 95% CI 1·02–2·98). The primary composite outcome had a coefficient of variation of 0·3 in the control group and 0·4 in the intervention group.

Most primary composite outcome events were due to death, loss to follow-up, treatment failure, or switch to multidrug-resistant tuberculosis treatment, which had similar percentages between the groups (203 events [14%; geometric mean] in 1350 patients in the control group *vs* 188 [13%] in 1283 in the intervention group; adjusted RR 0·97, 95% CI 0·69–1·35), and most of these were loss to follow-up on treatment (156 [10%] in the control group *vs* 133 [9%] in the intervention group; adjusted RR 0·92, 95% CI 0·59–1·44; table 2). In patients with a successful treatment outcome, recurrence rates were similar between the groups (1·5 recurrence per 100 person-years in the control group *vs* 2·4 per 100 person-years in the intervention group; unadjusted RR 1·60, 95% CI 0·75–3·42; table 2; appendix p 8).

Patients in the intervention group were 64% less likely to miss more than 20% of treatment doses in a month (geometric mean 0·9 months [16%] of 6·0 months per person in the intervention group *vs* 2·7 months [46%] of 6·0 months per person in the control group; adjusted RR 0·36, 95% CI 0·27–0·50) and missed 57% fewer doses (geometric mean 16 [11%] of 160 doses per person *vs* 42 [27%] of 160 per person; adjusted RR 0·43, 0·34–0·53) than those in the control group (table 3).

**Table 3:** Secondary outcomes of medication adherence and process measures

		**Control group**	**Intervention group**	**Unadjusted mean difference (95% CI)**	**Adjusted mean difference (95% CI)**
Adherence*					
Months in which patient missed >20% of doses per person per months of treatment		2·7/6·0 (46%)	0·9/6·0 (16%)	0·34 (0·24 to 0·49)	0·36 (0·27 to 0·50)
Doses missed per person per doses expected		42/160 (27%)	16/160 (11%)	0·40 (0·31 to 0·53)	0·43 (0·34 to 0·53)
Late or missed clinic visits per person per scheduled visits		2·6/5·0 (51%)	2·5/5·0 (49%)	0·96 (0·85 to 1·08)	0·97 (0·87 to 1·08)
**Process measures**					
Medication monitor error days per treatment months (rate per treatment month)		2429/7097 (0·2)	2812/6900 (0·4)	1·77 (0·88 to 3·56)	1·75 (0·91 to 3·36)
Quick openings per treatment months (rate per treatment month)		48 289/6987 (6·7)	62 309/6771 (9·0)	1·33 (1·07 to 1·66)	1·30 (1·06 to 1·60)
Withdrawals from use of medication event reminder monitor		105/1388 (6%)	106/1298 (8%)	1·27 (0·70 to 2·33)	1·26 (0·72 to 2·23)
Unable to use fixed-dose combination		79/1388 (5%)	46/1298 (4%)	0·72 (0·33 to 1·55)	0·75 (0·36 to 1·58)
Visits from township or village doctor per month					
	Participants included in analysis, n	1247	1132	..	..
	Mean (SD)†	2·5 (0·8)	2·2 (0·8)	−0·2 (−1·0 to 0·5)	−0·3 (−0·9 to 0·4)

Data are n/N (%) unless otherwise stated. All comparisons are intervention group versus control group.

* Adherence outcomes summarised by patient, taking the arithmetic mean within cluster, then the geometric mean between clusters (1306 in the control group and 1261 in the intervention group contributed to the analysis of adherence outcomes).

† Before any change in management in the intervention group.

Post-hoc sensitivity analyses for the primary outcome and secondary adherence outcomes were consistent with the primary analyses (appendix pp 8–10). The risk of primary composite outcome events increased with decreasing percentage adherence, by study group (appendix p 10).

Most patients in the control group reported self-administering treatment (943 [68%] of 1388 patients), and only 10% (138) were supervised by a health-care worker, although this differed widely by cluster (table 1; appendix p 5).

Before any change in patient management in the intervention group, patients in both groups had similar contact with the township and village doctor between clinic visits (mean 2·5 visits [SD 0·8] per month in the control group *vs* 2·2 visits [0·8] per month in the intervention group; adjusted mean difference –0·3, 95% CI –0·9 to 0·4). In the intervention group, intensive management was required for 196 (16%) of 1261 patients and was reported as received by 156 (82%) of 190. However, after patients were switched to intensive management, there was no reported increase in township or village doctor contact (mean 1·8 visits [SD 1·0] in the preceding month). Switching to directly observed therapy was required for 100 (8%) of 1261 patients and was reported as received by 53 (54%) of 99. Overall, 8% of patients in both groups withdrew from using the medication event reminder monitor (appendix p 11).

## Discussion

In this large, cluster-randomised trial of 2686 patients with drug-sensitive tuberculosis from four prefectures in China, a digital adherence technology intervention had no effect on the risk of the primary composite outcome, and secondary outcomes of death, treatment failure, or loss to follow-up during treatment. Tuberculosis recurrence was uncommon, with a 12-month risk of 1·9% after the end of treatment among patients who had a successful treatment outcome. Non-adherence was reduced by 57–64% in the intervention group compared with the control group, depending on the metric used, which was a greater reduction than in our previous study.^6^ A systematic review of digital adherence technologies to improve tuberculosis treatment outcomes reported intervention effects in different directions.^16^ In this study, the majority of patients in the control group self-administered treatment. Despite self-administration being a choice by the patient in consultation with their doctor, on the basis of national tuberculosis programme guidelines, the type of treatment support varied widely by cluster, suggesting it largely depended on the doctor's preference.

The intervention had no effect on loss to follow-up, which accounted for most of the primary composite outcome events (289 [67%] of 433 events); this might have been because there was a paucity of timely adherence data, and because of failure to change management following identification of non-adherence at monthly reviews. Rather than monthly adherence assessment, a more frequent review of adherence data by health-care workers and initiation of intensive management to assist patients who have issues with adherence are likely to be needed to reduce loss to follow-up. The Keheala intervention, evaluated in a study in Kenya, assessed weekly motivational messages, daily text message reminders, an unstructured supplementary service data platform for patients to confirm daily adherence with follow-up by the research team for patients who had not confirmed adherence, and clinic notification of patients with no confirmation for more than 2 days. The intervention was associated with a 68% (95% CI 50–79) reduced risk of poor treatment outcome (composite outcome of on-treatment death, treatment failure, or loss to follow-up), entirely through reducing loss to follow-up.^8^ A stepped-wedge trial in Uganda showed improved successful treatment outcomes (cured or completed treatment), including a reduction in loss to follow-up, among a per-protocol population who enrolled onto the intervention within the first 2 months of treatment.^9^ The study assessed an SMS-based intervention (99DOTS), whereby patients received daily text message dosing reminders and were asked to confirm when a dose was taken using a toll-free number, as well as a weekly automated interactive voice response check-in. Review of adherence data at visits every 2 weeks or monthly resulted in differentiated management based on adherence. Overall, 97% of patients in the control group and 52% in the intervention group were included in the per-protocol population. The authors acknowledge that this comparison might be problematic because of selection bias. An individually randomised trial in Peru, which used a real-time medication event reminder monitor showed, in the per-protocol population, higher treatment success (cured or completed treatment) in the intervention group than in the control group (48 [98%] of 49 *vs* 45 [85%] of 53; RR 1·15, 95% CI 1·02–1·30). However, this effect was not observed in the intention-to-treat population (RR 1·09, 95% CI 0·94–1·27), which included four patients in the intervention group who withdrew (two voluntarily [one switched to a different regimen], and two due to suspected misuse of the monitor).^10^ There was a failure to change patient management following identification of non-adherence at monthly reviews by doctors at the tuberculosis centres, despite regular visits to clusters by the national or county-level CDC to review intervention fidelity.

In our trial, despite non-adherence being higher in the control group than in the intervention group, there was no apparent intervention effect on treatment outcomes or tuberculosis recurrence, which contradicts the findings from some other studies. An analysis of the fluoroquinolone treatment trials, albeit a non-randomised comparison, showed a strong relationship between lower adherence and increased risk of unfavourable tuberculosis treatment outcomes.^3^, ^17^ This might mean that, in the control group, treatment adherence was still high enough to result in the majority of patients being effectively treated and having no increased risk of recurrence.^18^ With the granular adherence data generated by this trial, it will be important to identify whether certain patterns of non-adherence are associated with increased risk of poor treatment outcomes or treatment recurrence. In a descriptive analysis, our data showed a pattern of increased risk of unfavourable outcomes with lower adherence, in both groups, although confounding might partly explain this relationship. Alternatively, there might be differential measurement (between groups) of adherence using box-opening, resulting in adherence being similar in the two study groups. This would mean that box-opening is a poor proxy for treatment adherence. In a substudy of our previous trial, however, we did validate box-opening as a proxy using a urine test for rifampicin, and found high sensitivity and specificity.^19^ Future studies could include a validation of box-opening as a proxy of dose taken using urine testing to detect drug metabolites. We also cannot discount the Hawthorne effect of the silent-mode medication event reminder monitor box in the control group improving treatment adherence, compared with usual care.

Alternatively, we might have underestimated tuberculosis recurrence, although underestimation was unlikely to be differential by study group. We followed up patients for 12 months after the end of treatment, which would be likely to capture the vast majority of recurrences.^20^ Recurrence in this trial, however, was very low (1·9% over 12 months), in particular compared with the fluoroquinolone trials, where 12-month recurrence was two to three times higher than in this trial. We used solid culture in laboratories that had quality control assessed before, although not during, the study. Sputum specimens were only collected at two timepoints after end of treatment, limiting the measurement of recurrence, and specimens might have been of lower quality compared with those collected as part of a treatment trial. Our approach for documenting recurrence, therefore, might not have been sufficiently sensitive, although we did supplement sputum specimens with chest radiographs.

This trial did show a reduction in non-adherence, measured by box-opening, in the intervention group compared with the control group, similar to in our previous study,^6^ indicating improved quality of treatment with the intervention. Based on adherence data from these two pragmatic trials and programmatic experience of medication event reminder monitors in 138 counties in China,^21^ the China national tuberculosis programme has planned to expand the utilisation of the monitors, albeit with real-time functions, nationwide, in their 14th 5-year plan (for 2021–25).

Other digital adherence technologies have been assessed in upper-middle-income and high-income settings, including video-supported therapy, to improve treatment outcomes. These studies have shown similar or increased rates of favourable treatment outcomes with video supported therapy compared with control groups (often directly observed therapy); although, individually, all 95% CIs for the ratio effect estimate overlap 1, so are not significant.^22^, ^23^, ^24^ A study in China showed similar rates of successful treatment outcomes (cured and completed treatment) in the intervention group (video supported therapy; 109 [96%] of 203 patients) compared with the control group (directly observed therapy; 191 [95%] of 202).^23^ The observation in the intervention group was done using live video and directly observed therapy required observation of treatment by a health-care worker or lay worker once every 2 days. Further evaluation of this technology in China, where directly observed therapy is not a requirement, is warranted.

This trial has many strengths, including a large sample size and an intervention that was implemented by the national tuberculosis programme rather than a research team in parallel, it was conducted across varied settings, and follow-up continued for 12 months after the end of treatment. The study does, however, have several limitations: more intensive management activities, such as home visits to patients identified as having adherence problems, did not always happen as planned; after randomisation, two intervention clusters were combined, although this had minimal impact on power; solid culture rather than the more sensitive liquid culture was used to measure recurrence; and adherence outcomes were defined using a box-opening as a proxy for doses taken.

In conclusion, the digital adherence technology intervention, involving a daily reminder medication monitor, monthly review of adherence data, and differentiated care for patients with adherence issues, was inadequate to influence unfavourable outcomes, which included loss to follow-up during treatment, tuberculosis recurrence, death, and treatment failure. Poor implementation of targeted adherence support might have contributed to no effect of the intervention on outcomes. Other trials have shown that digital adherence technologies (SMS-based and those based on medication event reminder monitors), implemented in real-time, can improve successful treatment outcomes. It is important, though, to understand the cost of delivering such interventions and also how these can be implemented in routine practice. Programmatically, treatment adherence might be substandard and clinicians do not have adequate methods to measure dose-taking. WHO uses treatment success (cured or completed treatment) as an indicator for performance of tuberculosis programmes, although it is a poor indicator of care as treatment completion is often not based on robust measures of adherence. For evaluating digital adherence technologies, rather than relying solely on outcomes at the end of treatment, a combined endpoint of adherence and treatment outcome could be used, with adherence measured in all trial participants using digital technologies with excellent accuracy characteristics. It is important that future trials do measure end of treatment outcomes, ideally incorporating quality treatment completion and possibly recurrence, if measured robustly, to generate strong evidence to influence policy.

## Data sharing

The individual deidentified participant data that underlie the results reported in this article, data dictionary, study protocol, statistical analysis plan, and Stata code will be made available on London School of Hygiene & Tropical Medicine Data Compass, without restriction, immediately following publication.

## Declaration of interests

We declare no competing interests.
