# Supplementary material for: Digital adherence technologies to improve tuberculosis treatment outcomes in China: a cluster-randomised superiority trial
Source: Lancet Glob Health. 2023 Apr 14;11(5):e693–703. doi: 10.1016/S2214-109X(23)00068-2 (PMC10126227; doi:10.1016/S2214-109X(23)00068-2)
Supplement: Supplementary appendix [file mmc1.pdf]

THE LANCET
Global Health

Supplementary appendix

This appendix formed part of the original submission and has been peer reviewed. We post it as supplied by the authors.

Supplement to: Liu X, Thompson J, Dong D, et al. Digital adherence technologies to improve tuberculosis treatment outcomes in China: a cluster-randomised superiority trial. *Lancet Glob Health* 2023; **11:** e693–703.

Supplementary Appendix:

Digital adherence technologies to improve tuberculosis treatment outcomes: a cluster-randomised superiority trial

Xiaoqiu Liu, Jennifer Thompson, Haiyan Dong, Sedona Sweeney, Xue Li, Yanli Yuan, Xiaomeng Wang, Wangrui He, Bruce Thomas, Caihong Xu, Dongmei Hu, Anna Vassall, Shitong Huan, Hui Zhang, Shiwen Jiang, Katherine Fielding, Yanlin Zhao

Table of Contents

1. Effectiveness methods 2
  1.1. Randomisation 2
  1.2. Measurement of outcomes 2
  1.3. Statistical methods 3
  1.4. Protocol clarification 4
2. Effectiveness Results 4
  2.1. Characteristics of the trial sites and participants 4
  2.2. Per-protocol population 4
  2.3. Process measures 4
  2.4. Supplementary effectiveness figures 5
  2.5. Supplementary effectiveness tables 5
  2.6. Supplementary tables for treatment adherence 9
  2.7. Supplementary table for process measures 10

1. Effectiveness methods

1.1. Randomisation

The restriction criteria were defined as follows:

1. There are four prefectures (six in Hangzhou, seven in Weizhou, four in Jilin and seven in Ganzhou) in three provinces (13 in Zhejiang, 4 in Jilin and 7 in Jiangxi). The difference in the number of intervention and control group clusters should be at most one in each prefecture (or province).

2. There are 14 clusters with a designated hospital and 10 with a TB dispensary. There should be equal numbers of clusters with a designated hospital in each study group (and therefore equal numbers with a TB dispensary).

3. There are 7 urban clusters and 17 rural clusters. The difference in the number of intervention and control group clusters should be at most one in each of the urban and rural clusters.

4. The difference in the average number of smear-positive TB cases notified by the TB clinic in 2015 in each cluster between intervention and control group clusters is at most 10 cases.

The process for restricted randomization followed three steps:

1. Generate 10,000 random allocations and determine the “restriction factor” for each of the four criteria above. So long as at least 1% of allocations are eligible (giving at least 27,042 eligible allocations) then proceed to step 2. 1.24% of allocations were eligible.

2. If no restrictions or stratifications are applied, the probab7ility that any two clusters are in the same group should be 11/23 = 0.478. To ensure that valid statistical inferences can be drawn, we want to ensure that the probability that any two clusters are in the same group is not too far from 0. 478. For example, if the probability that two clusters are in the same study group is close to 1, then these two clusters may need to be treated as one cluster. Hence, the next step is to check these probabilities for all pairwise combinations (23*22/2 = 253 possibilities). This was done by simulating 5,000 acceptable allocations and then estimating the pairwise probabilities from these 5,000 allocations. The proportion where a pair of clusters were in the same study group varied between 0.288 and 0.745, which suggests that the most restrictive criteria still allows for appropriate statistical inference. Hence, these criteria were finalised for the randomisation.

3. One of the 5,000 can be chosen as the final allocation of the 24 clusters to the two groups. This was chosen using a random number generated by Stata. The random number seed to determine this was selected before any of the above steps were initiated.

All clusters were enrolled before this process began. Randomisation was conducted by James Lewis, the trial statistician at the time.

1.2. Measurement of outcomes

The treatment outcomes are based on standard NTP recorded outcomes. Poor end of treatment outcome is defined as treatment failure, died, developed multidrug resistant tuberculosis, lost-to-

follow-up, or stopping treatment due to an adverse reaction or refusal of treatment). Treatment failure (as documented on the TB register) is defined as positive sputum at month 5 or later, after treatment start) and on-treatment lost to follow-up (as documented on the TB register) is defined as patient whose treatment was interrupted for 2 consecutive months or more.

1.3. Statistical methods

All analyses used a cluster-level analysis containing all clusters. For this, the outcomes were summarised to a mean, proportion, or rate for each cluster. Our primary comparison for binary and rate outcomes were ratios. To compare the study groups, we look logarithms of the cluster summaries and compared the study groups using a t-test. The exponential of the mean difference in log-summaries gave a risk or rate ratio. For the absolute difference between the study groups, the clusters summaries were compared without taking logarithms. Adjustment was done using a two stage approach. First, a model was fit with the outcome as the dependent variable and adjustment covariates as the independent variables ignoring the intervention and clustering. This provided estimated residuals, which were summarised by cluster and analysed as described above.

<u>Multiple imputation</u>

For the primary composite outcome participants were classified as having a favourable outcome if: they had a treatment outcome at the end of treatment of cured or completed treatment and no recurrence defined by either a negative culture at 18 months or at 18 months, culture is missing, or the patient was unable to produce sputum and they have no signs of new active TB meaning (1) no self-reported restart of treatment or (2) no sign of new active TB on x-ray. The composite outcome was missing if a participant meets neither definition of a favourable or unfavourable outcome, particularly an issue if a patient has become lost to follow up after treatment completion, with no sign of a recurrence at their last follow-up visit.

For the primary outcome, multiple imputation was used to prevent bias arising from lost to follow up after completion of treatment. Chained equations were used to imputed poor treatment outcome, recurrence up to 12 months, and recurrence up to 18 months. Recurrence was defined by a positive culture at or before the designated time point, an x-ray indicative of TB, or a self-report of restarting treatment. Non-recurrence required a negative culture at the designated time. Otherwise, recurrence was missing and imputed. Components were simplified to good or poor treatment outcome, recurrence up to 12 months, or recurrence between 12 and 18 months. Imputations used logistic regression models by arm and adjusted for cluster and each of the other components. Data augmentation was used to overcome perfect prediction: this is where a small number of observations are added to the dataset to prevent the imputation model perfectly predicting the outcome. Imputation models included all adjustment covariates, cluster, and the two other components of the model. It was pre-specified that problematic covariates would be removed from the imputation. This resulted in a final set of imputation models that contained only cluster, and the other two component of a poor outcome.

Each imputed dataset was analysed with the cluster-level analysis method described above and the results were combined using Rubin’s rules

<u>Complete case analysis</u>

For the primary outcome, as a sensitivity analysis, a complete case analysis was also conducted. For all other outcomes, complete case analysis was the primary analysis. These analyses only included patients with a definable outcome.
For the primary composite outcome, classification of no TB recurrence was loosened so that a self-report of no restart of treatment, or a chest x-ray showing no signs of active TB at 18 months were sufficient for a patient to be classified as having a good composite outcome.

Subgroup analyses

For individual level characteristics, first calculate the risk of a poor outcome in each subgroup and cluster. Calculate the mean of these risks within subgroup to estimate the risk of a poor outcome in control and intervention groups. To test for a difference, calculate the difference between the two subgroups within each cluster. A t-test is performed on these differences to test for effect modification.
For cluster level characteristics, a linear regression will be used with study group, subgroup, and an interaction between the two as independent variables. A likelihood ratio test will be used to estimate a p-value for the interaction. The risk of a poor outcome and its confidence interval in each group will be calculated from the relevant coefficients from the regression.

1.4. Protocol clarification

There is inconsistency in the description of the frequency of Doctor visits for patients in the control arm in the protocol (Table 2 and appendix to the protocol). In the control arm of the trial the doctor from township health center visit TB patients every 10 days in the intensive phase, and once a month in the continuous phase. This is correctly described in Table 2 from the protocol.

2. Effectiveness Results

2.1. Characteristics of the trial sites and participants

Early in the trial, 19 patients were incorrectly counted as post-enrolment exclusions after enrolment due to misunderstandings of the trial protocols. Additional training was provided to trial sites, and these have been included in the intention to treat population despite missing values for most outcomes.

2.2. Per-protocol population

94.7% (1231/1300) and 93.4% (1156/1238) contributed to the per-protocol complete case analysis for the primary outcome, in the control (routine care) and intervention groups, respectively.

2.3. Process measures

The rate of medication event reminder monitor (MERM) malfunctions or errors was similar between the groups (0.2 and 0.4 days of treatment affected by errors per person-month in control and intervention groups respectively; adjusted rate ratio=1.75[0.91,3.36]). Patients in the intervention group were more likely to report non-use of the MERM due to travel (intervention arm 1.8% of days not using the intervention vs control arm 0.9% of days not using the MERM). Intervention group patients were 1.30(1.06, 1.60) time more likely than control patients to open the box for a short time that would not be long enough for this to signify taking treatment (<2 seconds). Length of

treatment was similar between the groups (mean 6 months control vs 6.1 months intervention, adjusted mean difference=+0.1[-0.3,+0.3]).

There was no difference between the groups in withdrawal from use of the MERM (control 105[GM 6%] vs intervention 106[GM 8%]); most of these were due to inability to use fixed dose combination therapy, which was also similar between the groups (control 79[GM 5%] vs intervention 46[GM 4%]).

2.4. Supplementary effectiveness figures

Figure S1: Adherence to treatment (measured by MERM) by month of treatment and study group

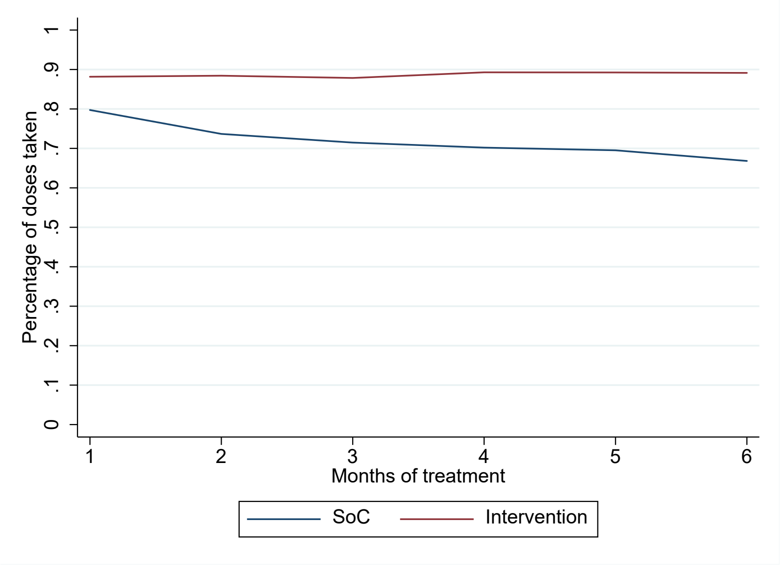

SoC standard of care (control)

Figure S2: Self-reported supervisor of medication, control arm

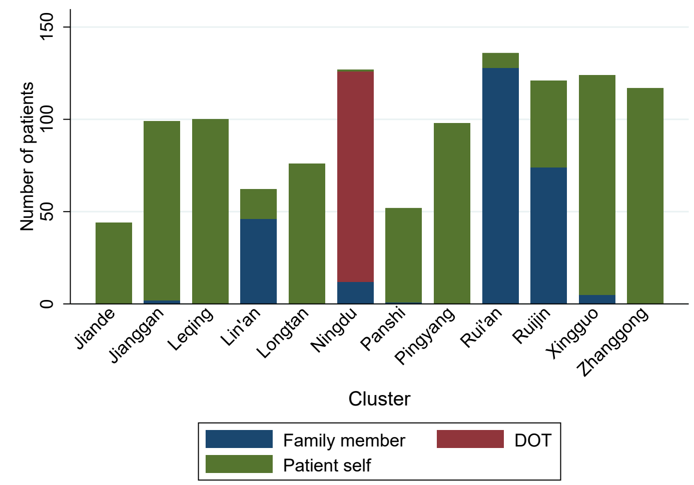

DOT directly observed therapy

2.5. Supplementary effectiveness tables

Table S1: Additional baseline characteristics of clusters

	Control	Intervention
**Cluster level covariate**	N=12	N=11
Number of townships, median(IQR)	17.5 (13.5-24.5)	21 (14-25)
Number of villages, median(IQR)	289.5(190-451.5)	257(136-520)
Number of doctors seeing TB patients, median (IQR)	2.5 (1.5-4)	2(1-3)
Per capita net income (CYN), median(IQR)	27877.5 (13653-38439)	34087 (17232.5-39238)
Population size per 1000, median(IQR)	603.5 (497.0-859.4)	760.0 (439.2-1084.8)
Laboratory onsite, n(%)	12 (100%)	11 (100%)
X-ray onsite, n(%)	12 (100%)	11 (100%)
1 month of TB medication dispensed during intensive phase, n(%)	12 (100%)	11 (100%)
1 month of TB medication dispensed during continuation phase, n(%)	12 (100%)	11 (100%)
Are incentives offered, n(%)	2 (17%)	1 (9%)
**Individual level covariate**	**N = 1388**	**N=1298**
Number of household members, median (IQR)	4 (3 – 5)	4 (3 - 5)
Have sufficient money to cover costs, n(%)	1099 (79%)	1147 (88%)
Distance to TB clinic, median (IQR)	15 (5 – 30)	15 (5 – 28)
Distance to supervision facility, median (IQR)	2 (1 – 3)	2 (1 – 2.5)

IQR interquartile range; CNY Chinese Yuan; X-ray chest radiograph

Table S2: Summary of the primary composite outcome and components of the unfavourable outcome, by group

	Control		Intervention	
	**n**	**%**	**n (%)**	
**Total number of participants**	**1388**		**1298**	
Composite outcome missing (undefined)	88	6.3%	60	4.6%
**Composite outcome defined:**	**1300**		**1238**	
**Composite favourable outcome**[a]	**1083**	**83.3%**	**1022**	**82.6%**
**Composite unfavourable outcome**[a]	**217**	**16.7%**	**216**	**17.4%**
Poor end of treatment outcome[b]*:*	*203*	*93.5%*	*188*	*87.0%*
Lost to follow up during treatment	156		133	
Treatment failure	33		45	
Death (TB or non-TB)	12		10	
Switch to MDR	2		0	
Recurrence in those with a good treatment outcome[b]*:*	*14*	*6.5%*	*28*	*13.0%*
Positive culture	9		22	
X-ray indicative of active TB only	1		2	
TB retreatment only	2		3	
X-ray indicative of active TB and TB retreatment	2		1	

[a] percentage of those with outcome defined; [b] percentage of those with a composite unfavourable outcome; X-ray chest radiograph

Table S3: Cluster summaries of primary unfavourable outcome (poor end of treatment outcome or recurrence)

**Control**		**Intervention**	
Mean of imputations (%)	Complete case n/N(%)	Mean of imputations (%)	Complete case n/N(%)
8.2	6/93 (6.4)	8.3	11/138 (8.0)
9.5	8/82 (9.8)	9.1	7/80 (8.8)
11.9	12/106 (11.3)	10.2	8/79 (10.1)
13.0	17/134 (12.7)	11.7	14/114 (12.3)
15.4	22/137 (16.1)	15.8	23/144 (16.0)
17.0	11/64 (17.2)	17.4	25/142 (17.6)
18.5	26/147 (17.7)	19.5	24/120 (20.0)
19.0	29/153 (19.0)	19.9	30/150 (20.0)
22.4	33/151 (21.8)	22.4	16/72 (22.2)
22.9	10/53 (18.9)	28.6	44/155 (28.4)
23.4	24/115 (20.9)	31.5	14/44 (31.8)
29.1	19/65 (29.2)		

Table S4: pre-specified and post-hoc sensitivity analyses for the primary unfavourable outcome

	Control	Intervention	Unadjusted arm comparison (95% CI)	Adjusted arm comparison (95% CI)
**Risk difference [a]**				
Multiple imputation	239/1388 (18%)	224/1298 (18%)	0.2% (-6.4%, 6.7%)	0.7% (-4.5%, 5.9%)
Complete case unfavourable outcome	217/1300 (17%)	216/1238 (18%)	1.0% (-5.1%, 7.0%)	1.6% (-3.1%, 6.2%)
**Risk ratio (complete case, pre-specified, sensitivity analyses)**				
Intention to treat	217/1300 (16%)	216/1238 (16%)	1.03 (0.71, 1.51)	1.05 (0.78, 1.41)
Per-protocol	205/1231 (15%)	195/1156 (16%)	1.04 (0.69, 1.57)	1.06 (0.78, 1.45)
Excluding NTM	217/1300 (16%)	215/1237 (16%)	1.03 (0.71, 1.51)	1.05 (0.78, 1.40)
**Risk ratio (Post-hoc sensitivity analyses)**				
Setting treatment 9 months or long as poor outcome [b]	255/1304 (19%)	246/1242 (19%)	1.01 (0.74, 1.38)	1.02 (0.76, 1.36)
Setting unable to produce sputum as negative culture in multiple imputation [b]	239/1388 (16%)	224/1298 (16%)	0.99 (0.66, 1.48)	1.01 (0.73, 1.40)

All sensitivity analysis used complete cases and unless specified used intention to treat populations and were pre-specified in the analysis plan

[a] Percentages shown are arithmetic means of the cluster level risks

[b] Post-hoc: Not pre-specified in analysis plan

NTM Non-tuberculous mycobacteria; CI confidence interval.

Table S5: Summary of the secondary poor end of treatment outcome and components of the outcome, by group

	Control			Intervention		
	n		%	n (%)		
**Total number of participants**	**1388**			**1298**		
Treatment outcome missing (undefined)		38	2.7%		15	1.2%
**Treatment outcome defined:**	**1350**			**1283**		
**Good end of treatment outcome [a]**	**1147**		**85.0%**	**1095**		**85.3%**
Cured	693			652		
Completed treatment	454			443		
**Poor end of treatment outcome [a]**	**203**		**15.0%**	**188**		**14.7%**
Lost to follow up during treatment	156			133		
Treatment failure	33			45		
Death (TB or non-TB)	12			10		
Switch to MDR	2			0		

[a] percentage of those with outcome defined

2.6. Supplementary tables for treatment adherence

Table S6: Sensitivity analyses for secondary treatment adherence outcomes

	**Control**	**Intervention**	**Unadjusted risk ratio (95% CI)**	**Adjusted risk ratio (95% CI)**
Months in which patients missed >20% of doses including days patients reported not using MERM / Months of treatment per person (%)	2.7/6.0 (47%)	1.1/6.0 (19%)	0.42 (0.31, 0.57)	0.44 (0.34, 0.56)
Doses missed including days patients reported not using MERM mean /doses expected per person (%)	45/163 (28%)	20/165 (13%)	0.46 (0.36, 0.58)	0.48 (0.40, 0.59)
**Post-hoc**				
Months in which patients missed >20% of doses including LFTU as non-adherence / Months of treatment per person(%)	3.0 / 6.2 (48%)	1.2/6.2 (20%)	0.41 (0.30, 0.57)	0.43 (0.33, 0.56)
Doses missed including LTFU as non-adherence / Doses expected per person(%)	48/164 (30%)	23/165(15%)	0.50 (0.39, 0.64)	0.52 (0.42, 0.65)

MERM medication event reminder monitor; LTFU lost to follow-up

Adherence outcomes summarised by patient, taking the arithmetic mean within cluster, then the geometric mean between clusters

Table S7: Post-hoc adherence measures

	Control	Intervention
Patients that missed at least one dose n/N (%)	1300/1306 (>99%)	1244/1261 (99%)
Number of times doses missed per patient mean (sd)	44.9 (39.3)	17.4 (16.1)
Days to first missed dose mean (sd)	87.6 (55.8)	87.1 (56.5)
Patients that ever missed 2 or more consecutive doses n/N (%)	1149/1306 (88%)	870/1261 (69%)
Number of times patients missed 2 or more consecutive doses mean(sd)	7.3 (7.1)	2.5 (3.4)
Patients that ever missed 3 or more consecutive doses n/N (%)	987/1306 (76%)	471/1261 (37%)
Number of times patients missed 3 or more consecutive doses mean(sd)	4.1 (4.9)	0.8 (1.7)
Patients that ever missed 4 or more consecutive doses n/N (%)	828/1306 (63%)	296/1261 (23%)
Number of times patients missed 4 or more consecutive doses mean(sd)	2.5 (3.4)	0.4 (1.0)

Analysis ignores missing data in counting consecutive doses. Similar result were found if missing data prevented doses being considered consecutive. sd standard deviation

2.7. Supplementary table for process measures

Table S8: Association between percentage adherence (measured by box-opening) and composite unfavourable outcome or recurrence, by study group

Percentage adherence	Control – composite unfavourable outcome n(%)	Control – recurrence* n(%)	Intervention - composite unfavourable outcome n (%)	Intervention – recurrence* n (%)
**≥ 90%**	27/332 (8%)	3/299 (1%)	72/700 (10%)	17/645 (3%)
**70% - 90%**	52/421 (12%)	2/371 (1%)	96/446 (22%)	10/360 (3%)
**< 70%**	106/500 (21%)	9/403 (2%)	18/60 (30%)	1/43 (2%)

* among those with a those with good treatment outcome (cured/completed treatment)

Table S9: Additional process measures

	**Control**	**Intervention**
N	1351	1283
Months of treatment, mean (sd) [a]	6.1 (0.4)	6.1 (0.3)
Percentage on treatment ≥8 months n/N(% [b])	61/1180 (5%)	49/1140 (4%)
Patient ever receive > 1 months of drugs with agreement of doctor n/ total n (%[b])	265/1387 (6%)	89/1296 (4%)
[Intervention only]		
Times alarm sounded before opening, Mean (sd), N		1.4 (0.1), 1261
Required intensive management n/total n(%[b])		196/1261 (8%)
Received intensive management		156/190 (74%)
Visits from township/village doctor per month after change in management mean (sd)		1.8 (1.0)
Required DOT n/total n(%[c])		100/1261 (4%)
Received DOT n/total n(%[c])		53/99 [d] (51%)
Withdrew from using the MERM n (%)	105/1388 (8%)	106/1298 (8%)

[a] Unadjusted mean difference: 0.0 (-0.3, +0.4) p= 0.87; Adjusted mean difference: 0.0 (-0.3, 0.3) p=0.92. IQR interquartile range, sd standard deviation; DOT directly observed treatment.
[b] Among trial participants with a treatment outcome of cured, completed course, or failure; [c] Geometric mean; [d] missing for one patient
